# Ultrahigh High‐temperature Capacitive Energy Storage Via Proton Irradiation

**DOI:** 10.1002/advs.202522035

**Published:** 2025-11-30

**Authors:** Chenyi Li, Hanxiao Gao, Yutie Gong, Yen‐Ting Lin, Yuquan Liu, Yuanqi Wang, Lan Chen, Hangyao Wu, Ling Cheng, Yang Li, Yang Liu, Huamin Zhou

**Affiliations:** ^1^ State Key Laboratory of Material Processing and Die & Mould Technology School of Materials Science and Engineering Huazhong University of Science and Technology Wuhan Hubei 430074 China; ^2^ Guangdong HUST Industrial Technology Research Institute Guangdong Provincial Key Laboratory of Manufacturing Equipment Digitization Dongguan Guangdong 523808 China; ^3^ Department of Chemical Engineering and Materials Research Institute Pennsylvania State University University Park PA 16802 USA; ^4^ RICOTON Technology Co., Ltd. Xiangxiang Hunan 411400 China

**Keywords:** dielectric energy storage, ether bonds, high‐dielectric polymers, proton irradiation

## Abstract

Polymer‐based film capacitors with ultrafast rates are extensively used in modern electronics and electric power systems. Dielectric polymers typically exhibit a low dielectric constant, while their energy density at elevated temperatures is limited by drastically increased conduction. To address this challenge, it is reported that proton irradiation enables concurrently enhanced dielectric constant and energy storage properties at high temperatures. The combined atomic force microscopy‐infrared spectroscopy and first‐principles calculations reveal that proton irradiation facilitates local rotation of ether bonds in aromatic polymers, producing greatly increased local polar states leading to markedly improved polarizability while preserving dense chain packing. Consequently, an ultrahigh discharged energy density of 6.9 J cm^−3^ with an efficiency > 95% is achieved in irradiated poly(ether imide) at 150 °C, exceeding current dielectric polymers and nanocomposites. The results suggest an alternative postprocessing method toward rational design of high‐performance dielectrics for capacitive energy storage.

## Graphical abstract

Proton irradiation concurrently induces enhanced dielectric constant and breakdown field in aromatic polymers with ether bonds, which enables an ultrahigh discharged energy density of 6.9 J cm^−3^ at above the efficiency of 95% at 150 °C, exceeding current dielectric polymers and nanocomposites. These findings address the promise of irradiation in optimizing dielectric energy storage of high‐temperature dielectric polymers.

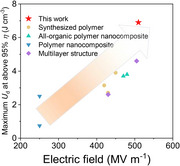

## 1 Introduction

Dielectric polymers exhibiting facile processability, high flexibility, great stability, and large breakdown strength are widely used in capacitive energy storage with ultrahigh power density.^[^ ^1^, ^2^, ^3^, ^4^, ^5^, ^6^, ^7^ ^]^ For linear dielectrics, the charged energy density is 1/2ε_*r*_ε_0_ *E* ^2^ (*E*, electric field, ε_*r*_, dielectric constant, and ε_0_, the vacuum permittivity). During the charge‐discharge process, the energy loss *U*_l_ arising from leakage or hysteresis loss usually occurs in dielectrics, giving rise to another parameter called charge‐discharge efficiency *η*. As a result, two key parameters, including discharged energy density *U*_d_ and efficiency *η* = *U*_d_/(*U*_d_+*U*_l_) are essential to evaluate the energy storage properties for electrostatic capacitor applications (Figure S1, Supporting Information). Most linear dielectric polymers show a relatively low ε_*r*_ of ≈2–3. For instance, the benchmark biaxially oriented polypropylene (BOPP) exhibits a low ε_*r*_ of ≈2 resulting in a low *U*_d_ of ≈3.5 J cm^−3^ at *E* = 600 MV m^−1^. To achieve ultrahigh *U*_d_ and *η*, it requires concurrently a high ε_*r*_ and a large breakdown field *E*_b_. However, these two parameters are usually inversely correlated through the relation *E*_b_−ε_*r*_ − 0.65.^[^ ^8^ ^]^ Moreover, *E*_b_ of BOPP decreases dramatically for temperatures above 100 °C owing to largely enhanced conduction, limiting its applications in harsh environments up to 150 °C (i.e., the inverter modules near combustion engine of hybrid electric vehicles). Consequently, there is an ever‐growing demand for next‐generation dielectrics exhibiting simultaneous enhancement of ε_*r*_ and *E*_b_ at high temperatures.^[^ ^1^, ^2^, ^3^, ^4^, ^5^ ^]^

In this context, a wide variety of aromatic polymers have been extensively exploited for high‐temperature dielectric energy storage.^[^ ^9^, ^10^, ^11^, ^12^, ^13^, ^14^, ^15^, ^16^, ^17^, ^18^, ^19^, ^20^, ^21^, ^22^, ^23^, ^24^, ^25^, ^26^ ^]^ Despite high heat resistance associated with highly conjugated structure, the conjugation effect of the aromatic backbones usually leads to lowered bandgaps and increased carrier migration, which is the main cause of the conduction loss of aromatic polymers at elevated temperatures under high electric fields.^[^ ^27^ ^]^ To inhibit electrical conduction, various approaches including molecular engineering,^[^ ^9^, ^10^, ^11^, ^12^, ^25^, ^26^ ^]^ nanocomposite,^[^ ^18^, ^19^, ^20^ ^]^ and surface coating^[^ ^15^, ^16^, ^17^ ^]^ have been developed. For instance, recent works reported that the dihedral angles between neighboring aromatic rings are strongly related to the trap depth at elevated temperatures.^[^ ^12^, ^25^ ^]^ Through the *π–π* stacking interactions, 2D highly ordered arrays with coexistence of high thermal conductivity and low electrical conduction were designed in ladderphane copolymers, which gives rise to a *U*_d_ of 3.9 J cm^−3^ at *η* > 95% at 150 °C^[^ ^26^ ^]^ Despite reduced electrical conduction, ε_*r*_ remains low (≈3–4) in most works.^[^ ^1^, ^17^, ^18^, ^26^ ^]^ Interestingly, ε_*r*_ can be substantially enhanced in diluted aromatic polymer nanocomposites.^[^ ^24^, ^28^, ^29^, ^30^ ^]^ Although the physical origin remains elusive, recent atomic force microscopy‐infrared spectroscopy (AFM‐IR) provides insights that the strong variation in the ether linkage between two aromatic rings occurring near polymer matrix‐filler interfacial regions^[^ ^28^ ^]^ may be responsible for the largely improved ε_*r*_ observed in diluted aromatic polymer nanocomposites.^[^ ^24^, ^28^, ^29^, ^30^ ^]^ Consequently, the molecular design of aromatic rings in terms of the connecting dihedral angles and stacking morphology is essential to maintain concurrent improvement of ε_*r*_ and *E*_b_ in aromatic polymers at elevated temperatures.

In this work, distinct from previous compositional approaches,^[^ ^9^, ^10^, ^11^, ^12^, ^17^, ^23^, ^25^, ^26^ ^]^ proton irradiation is used as postsynthesis processing method to ease the bond rotation of the connecting ether between aromatic rings which gives rise to increased polar entities, improved polarizability, and thus large ε_*r*_. Meanwhile, large angular distortions may induce spatial arrangement of aromatic groups, resulting in reduced interchain spacing and a dense package of molecular chains with enhanced elastic modulus, which suppresses the conduction loss at high temperatures and therefore enhances *E*_b_. Our work shows that proton irradiation can bring a structure with largely suppressed conjugation effect in aromatic polymers, which enables an ultrahigh *U*_d_ of 6.9 J cm^−3^ at above 95% at 150 °C. Our work addresses the promise of irradiation in developing high‐performance dielectric polymers for capacitive applications.

## 2 Results and Discussion

### 2.1 Mechanism Using Proton Irradiation to Improve Energy Storage

**Figure 1:**
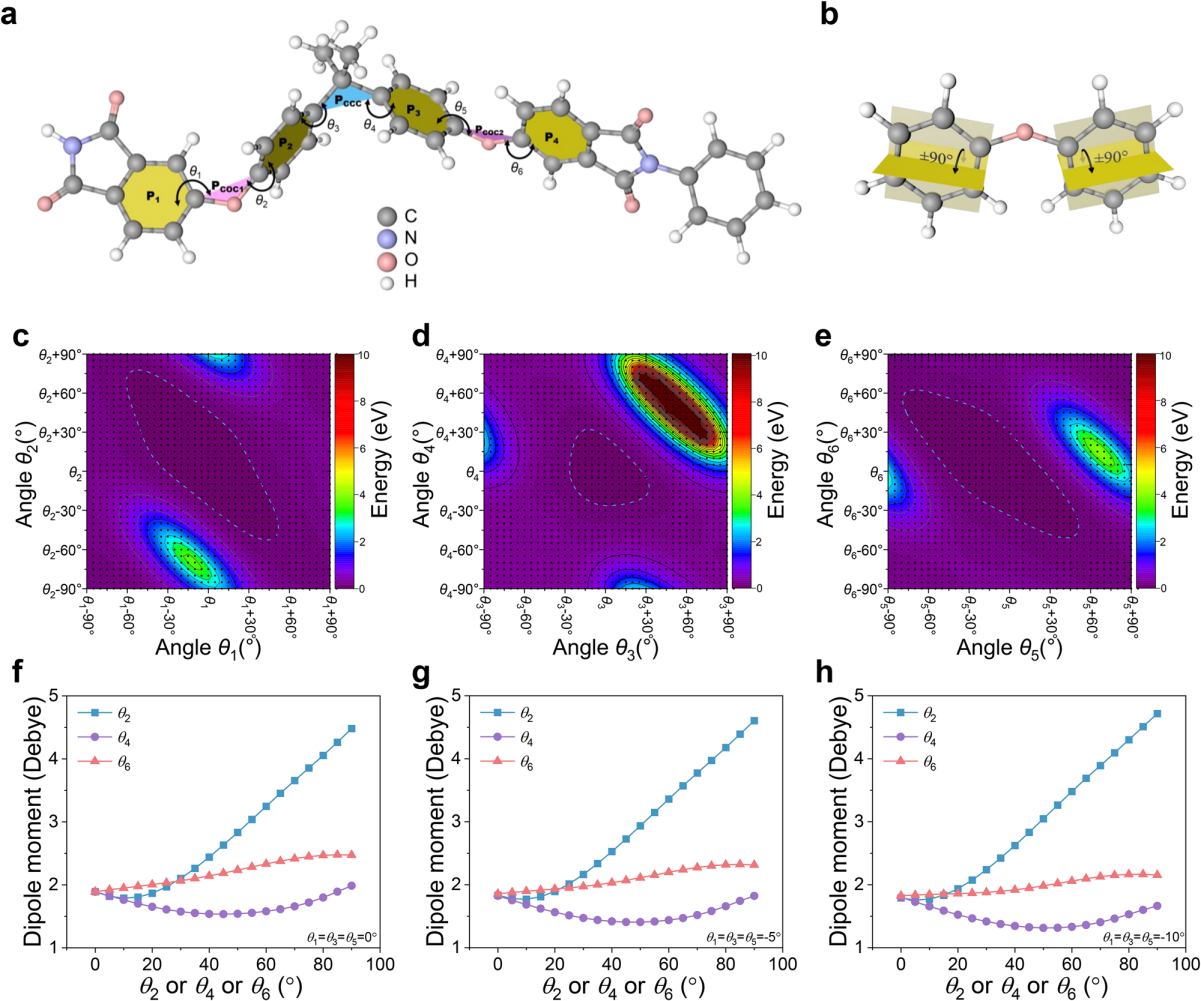
DFT calculations on the energy gain induced by local bond rotation. a) Sketch of the structure of PEI after relaxation. The dihedral angles *θ*_1_–*θ*_6_ between aromatic planes, including P_1_‐P_4_, P_COC1_, P_CCC,_ and P_COC2_ are indicated. b) Sketch of multiple polar states arising from the rotation of the C─O─C bond. To describe the increased number of polar states with a dihedral angle range from −90° to 90°, only two aromatic units were plotted. c) Calculated energy gain resulting from the rotations of *θ*_1_ and *θ*_2_. d) Calculated energy gain induced by the rotations of *θ*_3_ and *θ*_4_. e) Calculated energy gain due to the rotations of *θ*_5_ and *θ*_6_. f) Dipole moment under varied *θ*_2_, *θ*_4_, and *θ*_6_ (*θ*_1_ = *θ*_3_ = *θ*_5_ = 0°). g) Dipole moment under varied *θ*_2_, *θ*_4_, and *θ*_6_ (*θ*_1_ = *θ*_3_ = *θ*_5_ = −5°). h) Dipole moment under varied *θ*_2_, *θ*_4_, and *θ*_6_ (*θ*_1_ = *θ*_3_ = *θ*_5_ = −10°).

In this work, poly(ether imide) (PEI), one of the best high‐temperature dielectric polymers, is used for irradiation study as it contains two ether bonds in its chemical structure (**Figure** **1**a; Figure S2, Supporting Information). Using density functional theory (DFT) calculations, we compute the energy gain induced by the rotation of the dihedral angles between aromatic rings (Figure 1a). The dihedral angle between aromatic groups is defined as the angle between one specific aromatic plane with either the C‐C‐C plane or the C‐O‐C plane (Figure 1a).^[^ ^28^ ^]^ This definition mainly focuses on the local angular distortions is different from that used in previous work using the average values of all the dihedral angles of adjacent conjugated planes in a repeated polymer unit.^[^ ^12^, ^25^ ^]^ Interestingly, the calculated results show the ease of rotation with the C─O─C (or ether) bond as the linkage (Figure 1b,c,e) compared to that with the C─C─C bond (Figure 1d), which generally requires less energy to trigger the same angular distortions. These results imply that only weak local angular distortions may occur with a limited number of polar entities in pristine PEI where the conduction loss mainly arises from the conjugated segments. Moreover, via proton irradiation acting as an energy input, substantially more local polar entities in terms of the states with varied angular distortions may be created with larger distorted rotation angles (Figure 1b). This may lead to geometric reconstruction, which helps to break the conjugation effect inherent to most aromatic polymers. This is also different from the results obtained in the diluted PEI‐based nanocomposites, as the large angular distortions in ether bonds only occur near the interfacial regions (a few to tens nm) rather than the whole polymer matrix.^[^ ^28^ ^]^ The change in chain morphology in amorphous polymers is essential to tune the interchain charge transport. In this regard, our DFT results mark the foundation for future exploration combining deeper theoretical insights and advanced structural characterization. Moreover, the dipole moments as a function of angular distortions are further calculated (Figure 1f–h; Figure S3, Supporting Information). In our calculations, the linking dihedral angles, including ether bonds (i.e., *θ*_2_, *θ*_6_) and the other type (*θ*_4_) are varied, where *θ*_1_, *θ*_3_, *θ*_5_ are fixed at 0°, −5°, −10°, and −15° to simplify the calculations. Interestingly, the large increase in the dipole moment is achieved for rotating the ether bonds (i.e., *θ*_2_, *θ*_6_) exceeds that obtained by rotating the other type of connecting angles (*θ*_4_) for different cases considered in this work (Figure 1f–h; Figure S3, Supporting Information). The largest enhancement occurs for the rotation in *θ*_2,_ especially at high rotating angles (Figure 1f–h; Figure S3, Supporting Information). These findings, therefore, explicitly correlate the local angular distortions with the enhanced dipole moments, which provide insights into the enhanced dielectric constant and polarizability triggered by local distortions in the ether bonds due to irradiation.

The irradiation‐induced angular distortions in ether bonds may result in simultaneously enhanced ε_*r*_ and *E*_b_. As proton irradiation may result in ether bond rotation, bringing a large number of polar states resulting in a pronounced increase in the total number of polar entities (Figure 1b), irradiated aromatic polymers containing ether bonds may exhibit greatly enhanced dielectric polarizability, which benefits a large ε_*r*_. The quantitative relation between local polar entities and ε_*r*_ is beyond the scope of this work, requiring efforts in future studies. Meanwhile, the rotation of ether bonds driven by proton irradiation may help to weaken the conjugation effect through the formation of non‐planar segments, enabling the self‐assembly of highly dense chain packing. This may further enhance *E*_b_ owing to the reduced voids and/or free volume.^[^ ^28^ ^]^ Consequently, proton irradiation may provide an alternative route to regulate the energy storage properties at high temperatures by unveiling a previously unidentified high‐dielectric phase.

### 2.2 Formation of High‐Dielectric and High‐Insulating State

**Figure 2:**
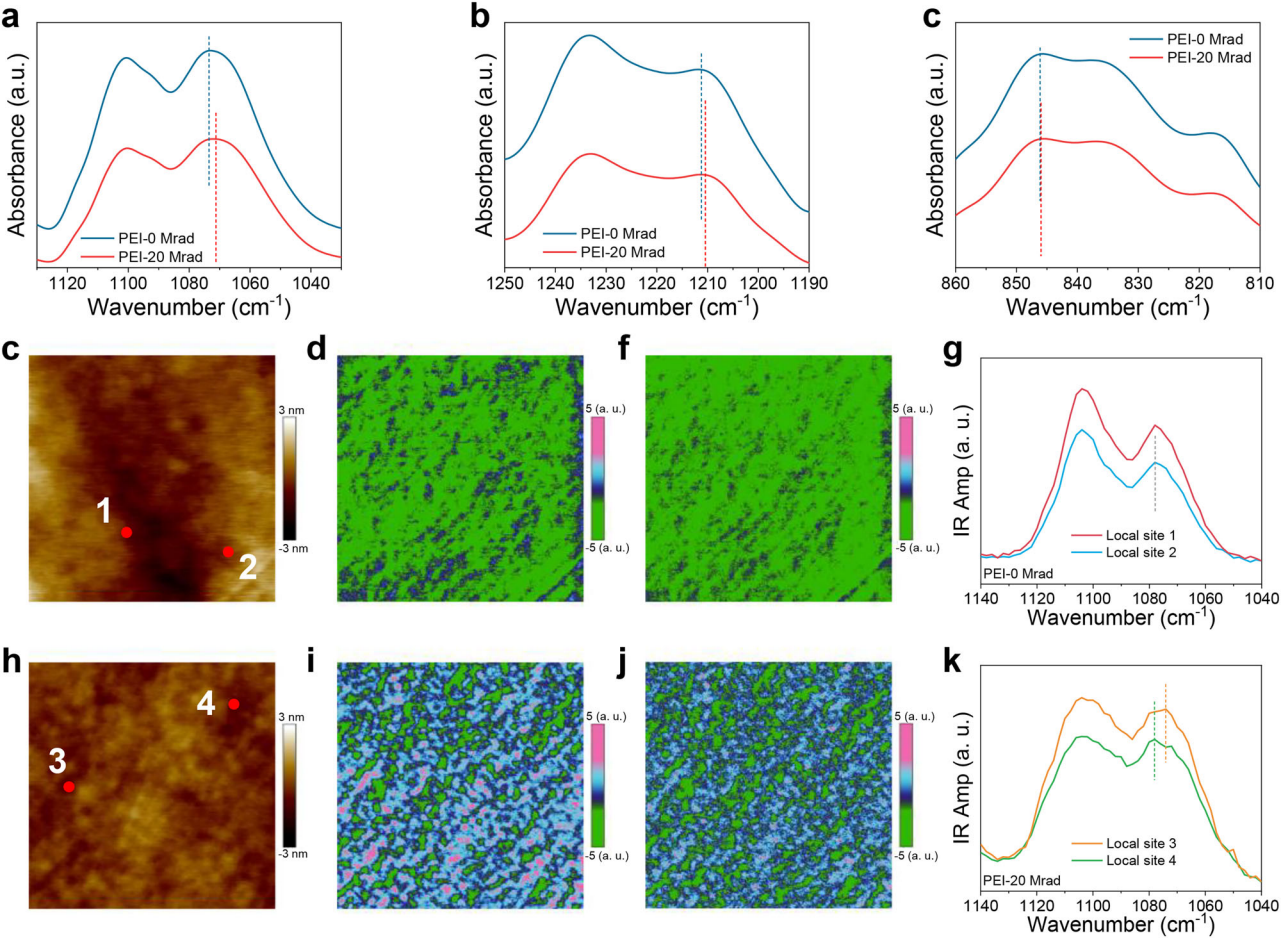
Characterization of irradiated PEI using FTIR and AFM‐IR. a–c) FTIR spectra. a) Infrared band at ≈1073 cm^−1^ indicative of symmetric stretching vibration of ether bonds. Irradiation causes a shift toward lower wavenumbers. b) Infrared band at ≈1211 cm^−1^ indicative of asymmetric stretching vibration of ether bonds. Irradiation causes a shift toward lower wavenumbers. c) The infrared band at ≈846 cm^−1^ is indicative of asymmetric stretching vibration of ether bonds and aromatic ring‐H stretching. Irradiation causes nearly no change in peak wavenumber. c–k) AFM‐ infrared results for x = 0 and 20 Mrad. c,h) Topography (1 × 1 µm^2^) for x = 0 and 20 Mrad. d,i) Chemical map irradiated with a 1073 cm^−1^ laser for x = 0 and 20 Mrad. f,j) Chemical map irradiated with a 1211 cm^−1^ laser for x = 0 and 20 Mrad. g,k) Local infrared spectra obtained from different sites in c and h. The dashed lines in a–c, g, and k, indicative of peak positions of infrared bands, are guides for the eyes.

Irradiated PEI has been evaluated by bulk Fourier‐transform infrared spectroscopy (FTIR) and AFM‐IR^[^ ^28^, ^31^, ^32^ ^]^ to provide deep structural insights into the formation of a high‐dielectric state. The FTIR wavenumber range used to analyze the structural changes of PEI before and after irradiation is chosen based on previous work.^[^ ^28^ ^]^ FTIR spectra (Figure S4, Supporting Information) clearly show the existence of irradiation‐induced wavenumber shift in characteristic infrared bands of ether bonds at ≈1073 cm^−1^ (symmetric stretching vibration of ether bonds, **Figure** **2**a), which is more visible than that at ≈1211 cm^−1^ (asymmetric stretching vibration of ether bonds, Figure 2b) and 846 cm^−1^ (asymmetric stretching vibration of ether bonds and aromatic ring‐H stretching, Figure 2c). These results suggest that proton irradiation and ether bonds are correlated, which may provide support for our design. We note that functional‐group vibrations exhibit a spectral range in bulk FTIR spectra. In this regard, the shift in infrared bands may not necessarily indicate the presence of proton‐induced change in ether bonds. Indeed, the presence of an infrared band shift was also observed in previous works on polymer nanocomposites, which was attributed to the change in the ether bonds near the interfacial region between nanofillers and polymer matrix.^[^ ^28^ ^]^ To further confirm the structural analysis made by bulk FTIR, other structural techniques are highly desirable.

As bulk FTIR provides structural analysis obtained on the relatively large scale (10–20 µm), the observation of small wavenumber shifts under irradiation remains challenging to identify (i.e., Figure 2a) as the band positions may correspond to a range in FTIR analysis. In this regard, characterization of distortions occurring in local bands is highly important, which may provide deeper insights that remain challenging to be implemented by bulk FTIR. To describe the high‐dielectric state at the molecular level, AFM‐IR was performed on the surface of pristine and irradiated PEI (Figure 2c,h). The typical chemical mapping results show that both symmetric and asymmetric stretching modes of ether bonds remain nearly homogeneous in pristine PEI (Figure 2d,f). The local infrared spectra obtained from different locations display a sharp infrared peak at near 1078 cm^−1^ corresponding to symmetric stretching of ether bonds, which shows the absence of wavenumber shift (Figure 2g). The presence of even larger band shift revealed by AFM‐IR (Figure 2k) than the counterpart obtained by bulk FTIR (Figure 2a) further confirms the presence of local heterogeneity critically related to the ether bonds, which can be greatly modified by proton irradiation. While AFM‐IR provides structural insights into local heterogeneity, Kelvin probe force microscopy (KPFM) may offer additional evidence of the potential change arising from the local disorder. Such local disorder arising at the nanoscale bond variation present in amorphous polymers remains challenging to be directly analyzed by XRD methods such as wide‐angle X‐ray scattering. Additional evidence obtained by other techniques is highly desirable in future studies as they may help substantiate the presence of chemical or conformational disorder based on the rapid development of experimental characterization techniques.

Note that the local spectra are noisy and show artifacts (e.g., a small peak at ≈1050 cm^−1^ in position 4 in Figure 2k). More representative and smoother spectra from multiple locations are desirable in future characterization studies using the AFM‐IR technique. Meanwhile, the AFM‐IR results mainly probe the surface through the photothermal effect, whereas the typical probing depth can exceed 500 nm. The proposed structural changes (e.g., ether bond rotation) occurring throughout the bulk of the material can be further verified in future AFM‐IR studies on the cross‐section of polymer films. Our primary result is consistent with our DFT calculations, revealing that pristine PEI without modifications adopts a rigid structure with weak angular distortions in ether bonds (Figure 1b,d). By contrast, the irradiated PEI displays a disordered chemical mapping manifesting the formation of a high‐dielectric state (Figure 2i,j). Infrared band shift behavior has also been observed in PEI‐based diluted nanocomposites owing to the interface effect,^[^ ^28^ ^]^ which only shows disordered chemical patterns near the interfacial regions in contrast with our results. Moreover, the local infrared spectra provide further evidence showing the broadness ofthe infrared band with a noticeable wavenumber shift (Figure 2k) owing to the formation of a great number of polar states with various angular distortions in ether bonds. Consequently, our structural results reveal the crucial role of irradiation‐induced modifications of the ether bonds in driving the formation of high‐dielectric PEI.

**Figure 3:**
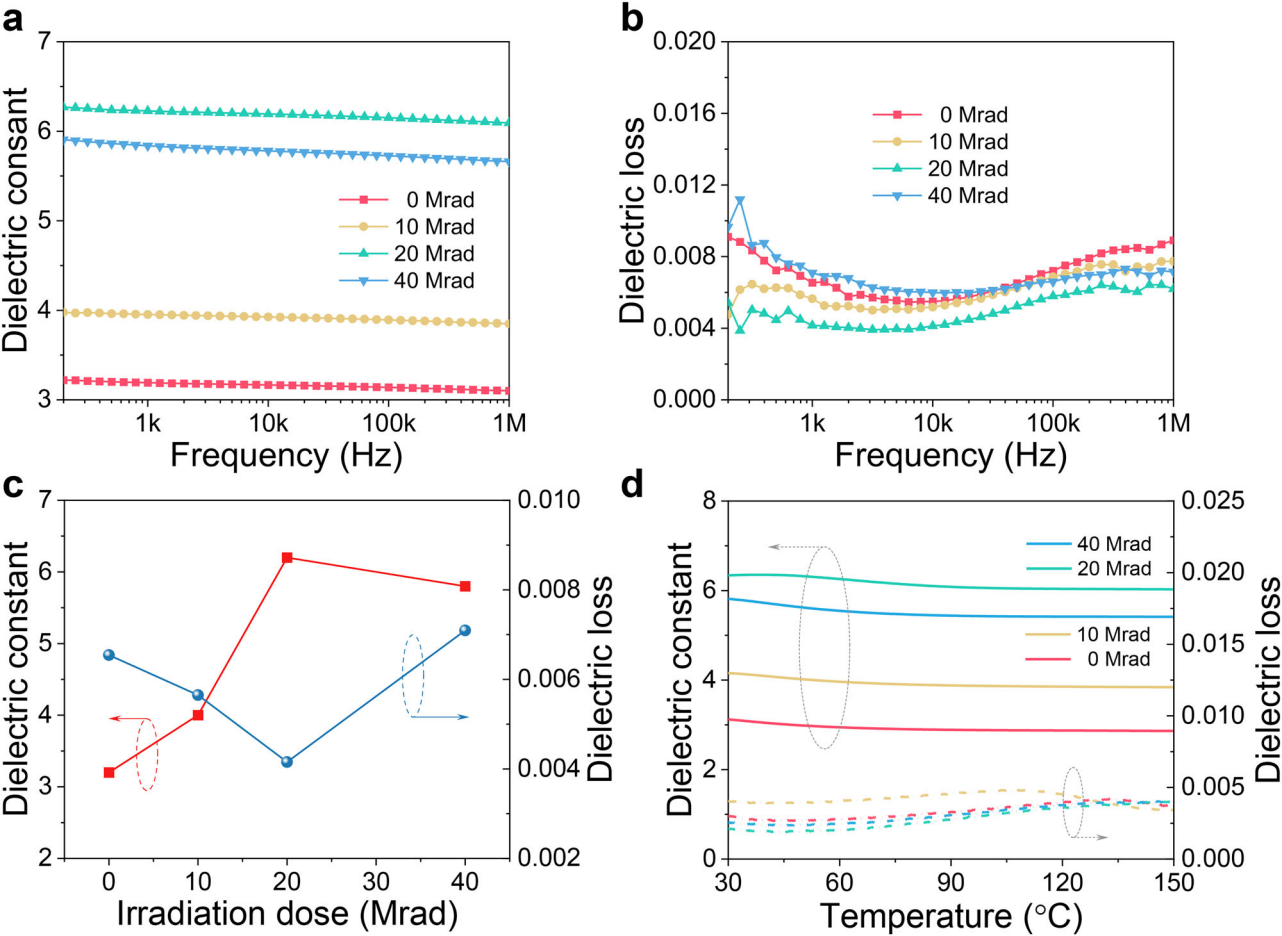
Dielectric properties of irradiated PEI. a) Frequency dependence of dielectric constant with different irradiation doses measured at room temperature. b) Frequency dependence of dielectric loss with different irradiation doses measured at room temperature. c) Summary on dielectric constant and loss at 1 kHz as a function of irradiation dose at room temperature. d) Temperature dependence of dielectric constant and loss at 1 kHz.

**Figure** **3**a,b present the frequency dependence of ε_*r*_ and dielectric loss under different irradiation doses *x*. Interestingly, Figure 3c shows that ε_*r*_ exhibits a marked increase from 3.2 at *x* = 0 Mrad to 6.2 at *x* = 20 Mrad followed by a plateau while dielectric loss reaches its minimum at *x* = 20 Mrad. This corresponds to ≈90% enhancement in ε_*r*_, which exceeds those obtained by typical molecular engineering approaches reported in pure dielectric polymers^[^ ^12^, ^17^, ^18^, ^23^, ^25^, ^26^ ^]^ (Table S1, Supporting Information), demonstrating the promise of irradiation as a useful postprocessing method in energy storage applications. The temperature dependence of ε_*r*_ and dielectric loss are depicted in Figure 3d which shows that the dielectric properties in irradiated PEI remain stable up to 150 °C. Our dielectric data therefore provide support on the appearance of irradiation‐induced high‐dielectric state in PEI.

**Figure 4:**
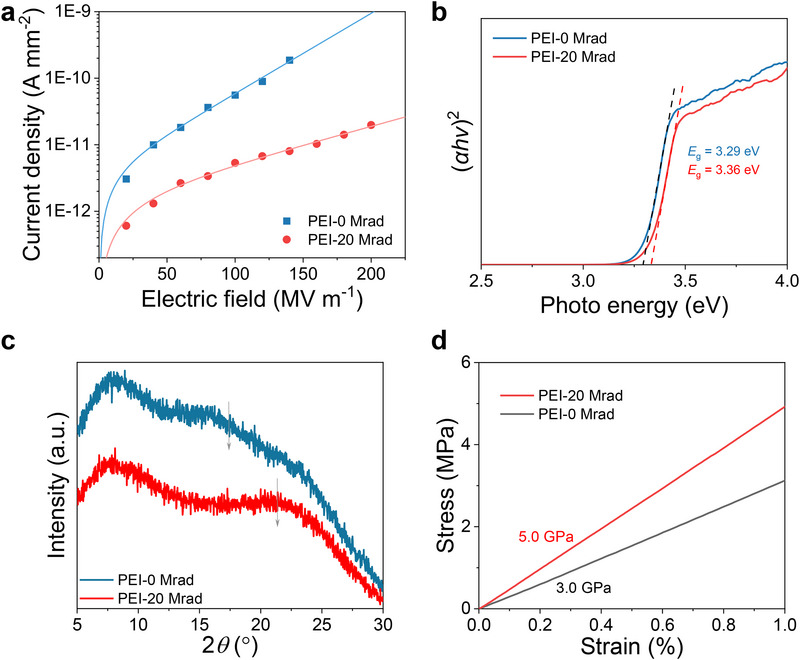
Reduced conduction in irradiated PEI at high temperatures. a) Leakage current measured at 150 °C. b) Bandgap *E*_g_ deduced from UV–vis results. *E*_g,_ values are shown. c) *θ*‐2*θ* XRD scans. The gray arrows are indicative the peak position of the broad peak. d) Stress–strain curves.

Electrical conduction at high temperatures is another critical parameter to evaluate the high‐temperature energy storage. The conduction results show that postprocessing of aromatic polymers with ether bonds by irradiation leads to remarkably reduced electrical conduction at elevated temperatures (**Figure** **4**a), which is desirable for high‐temperature energy storage applications. The fitting of leakage current density confirms that hopping conduction is the dominant mechanism in both pristine and irradiated PEI (Figure 4a). Hopping distance *λ* characteristic of the typical distance for electrons to hop between different trap sites decreases notably from 2.02 nm at *x* = 0 Mrad to 0.94 nm *at x* = 20 Mrad, indicating greatly increased trap depth. Thermally stimulated depolarization current (TSDC) results depicting the detrapping of charge carriers show that peak current occurs at much higher temperature (172.4 °C for *x* = 0 Mrad vs 215.8 °C for *x* = 20 Mrad) with a much sharper and higher peak current value (Figure S5, Supporting Information), which collaboratively supports the enhanced trap depth driven by proton irradiation. These results are also consistent with previous work displaying the positive correlation between the trap depth and the dihedral angle between adjacent benzene rings.^[^ ^25^ ^]^ In addition, the bandgap of PEI after irradiation shows a slight increase from 3.29 eV at *x* = 0 Mrad to 3.36 eV at *x* = 20 Mrad (Figure 4b), suggesting suppression of the conjugation effect of the aromatic groups induced by proton irradiation.

To provide structural insights into the lowered electrical conduction, we performed XRD *θ*‐2*θ* scans on pristine and irradicated PEI (Figure 4c). We show in Figure 4d that both pristine and irradiated PEI present very broad peaks owing to the amorphous nature. Interestingly, we observe that the broad peak at around 17.4° in pristine PEI shifts pronouncedly toward 21.4° in irradiated PEI, corresponding to a considerable reduction in interchain spacing from 0.51 to 0.41 nm. This result strongly suggests that proton irradiation enables the rotation of the dihedral angles, which may make polymer chains self‐assemble into highly dense packing. The dense chain packing of polymer chains caused by proton irradiation is also supported by markedly enhanced modulus from 3.0 GPa for *x* = 0 Mrad to 5.0 GPa for *x* = 20 Mrad (Figure 4d). The enhanced mechanical properties, in turn, help to inhibit the electromechanical breakdown, which is one of the dominant mechanisms for the dielectric breakdown.^[^ ^5^ ^]^

### 2.3 High‐Temperature Energy Storage

**Figure 5:**
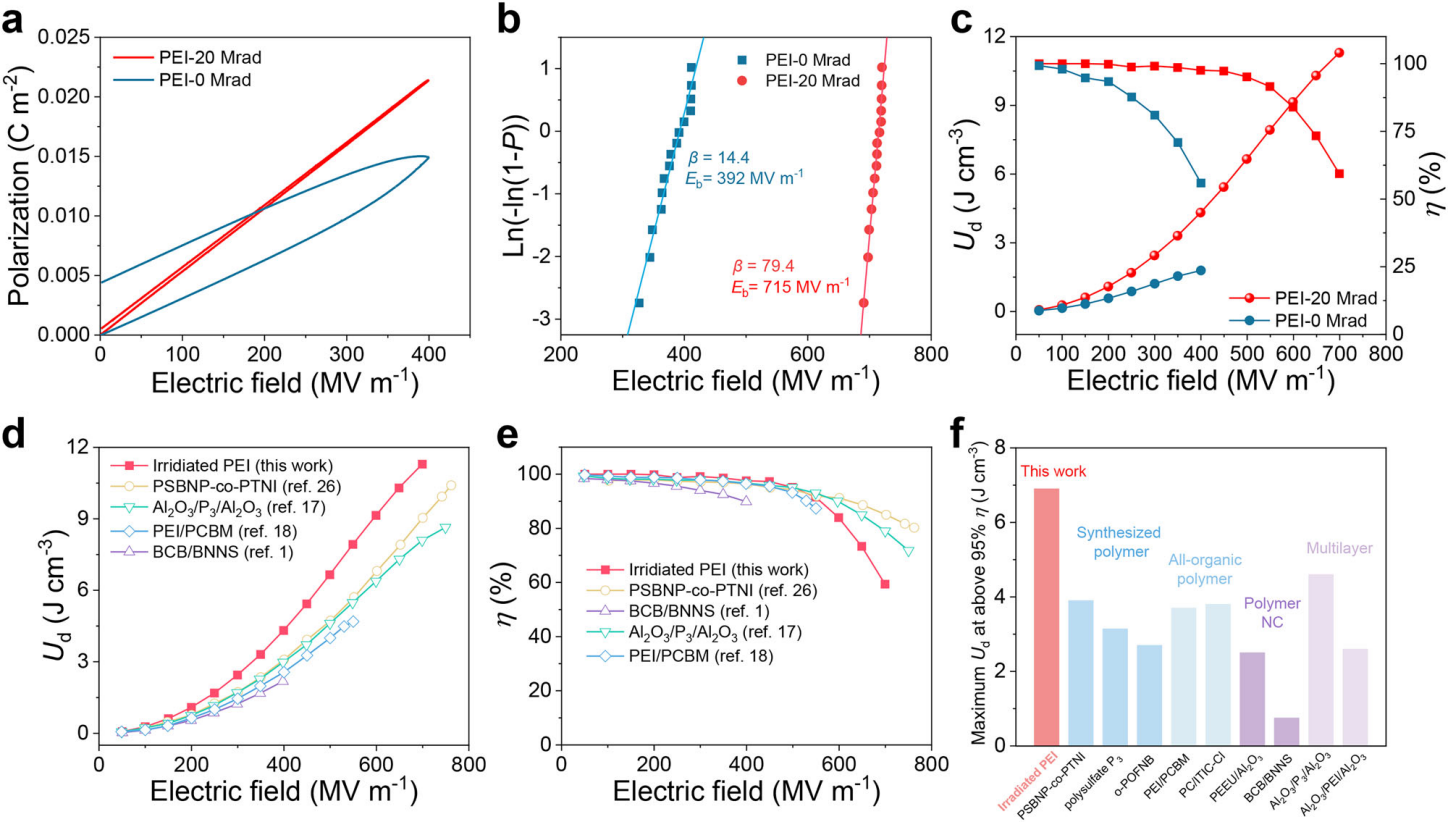
Energy storage of irradiated PEI. a) Unipolar *P*‐*E* loops measured at 150 °C and 100 Hz. b) Weibull breakdown strength. c) Discharged energy density and efficiency at 150 °C. d) Comparison of *U*_d_ between current work with previous ones at 150 °C. *P*_3_ indicates polysulfate. BNNS indicates boron nitride nanosheets. e) Comparison of *η* between the current work and previous results at 150 °C. f) Comparison of *U*_d_ at *η* > 95% between irradiated polymers and polymer‐based dielectrics at 150 °C. NC is short for nanocomposites.

We have presented that high‐dielectric design by proton irradiation enables simultaneous improvement in ε_*r*_ and heat resistance in aromatic polymers with ether bonds, which is beneficial to energy storage at high temperatures. Note that dielectric loss is usually measured under small electric fields (i.e., 1 V used in this work). The small dielectric loss does not necessarily indicate the presence of low hysteretic loss obtained by *P*‐*E* loops under much higher electric fields. Both losses are frequency‐dependent.^[^ ^1^ ^]^ For energy storage applications, the measurement frequency of 100 Hz was used in this work, which is consistent with recent studies.^[^ ^5^ ^]^ We therefore focus on energy storage behavior through typical *P*‐*E* loop measurements at 150 °C (**Figure** **5**a). Extremely slim *P*‐*E* loops with enhanced maximum polarization and nearly vanishing remanent polarization are observed in irradiated PEI, indicating a large polarization change (Figures S6–S9, Supporting Information), which is desired for energy storage applications.^[^ ^1^, ^2^, ^3^, ^4^, ^5^ ^]^ By contrast, *P*‐*E* loops in pristine PEI are lossy with large remanent polarization and hysteresis loss owing to the increased conduction at elevated temperatures (Figure 5a). The breakdown behavior is analyzed through the two‐parameter Weibull equation, which shows that proton irradiation results in a superior *E*_b_ of 715 MV m^−1^ (*x* = 20 Mrad) compared to that (392 MV m^−1^) of pristine PEI (Figure 5b).

Typical energy storage properties, including *U*_d_ and *η* are summarized in Figure 5c. Owing to the high conduction loss (Figure 4a), *U*_d_ at *η* > 95% is only ≈0.3 J cm^−3^ in pristine PEI. On the contrary, irradiated PEI (*x* = 20 Mard) displays an exceptionally high *U*_d_ of 6.9 J cm^−3^ at *η* > 95% under 510 MV m^−1,^ corresponding to ≈22 times improvement over the pristine counterpart. Our results also compete favorably with the state‐of‐the‐art results including the incorporation of high‐electron affinity organic semiconductors (i.e., phenyl‐C61‐butyric acid methyl ester (PCBM))^[^ ^18^ ^]^ with a *U*_d_ of 3.7 J cm^−3^ at *η* > 95%, surface coatings of high‐insulating wide bandgap inorganic thin layer (i.e., Al_2_O_3_)^17^ with a *U*_d_ of 4.6 J cm^−3^ at *η* > 95% and structural modifications by high thermal conductivity strategy^[^ ^26^ ^]^ with a *U*_d_ of 3.9 J cm^−3^ at *η* > 95% (Figure 5d,e; Tables S1 and S2, Supporting Information). Irradiated PEI therefore exhibits an outstanding *U*_d_ especially for the electric field below 550 MV m^−1^ (Figure 5d) as it shows considerably higher ε_*r*_ than previous works.^[^ ^1^, ^17^, ^18^, ^26^ ^]^ Our results also show that proton irradiation as a postprocessing strategy can achieve a larger *U*_d_ at *η* > 95% than synthesized polymers, all‐organic polymers, polymer nanocomposites, and multilayer polymers (Figure 5f; Tables S1 and S2, Supporting Information). Consequently, our work demonstrates the great potential of irradiation strategy for the optimization of high‐performance dielectric polymers for high‐temperature energy storage applications.^[^ ^21^, ^34^, ^35^, ^36^ ^]^

### 2.4 Discussions

Our work suggests that the linkage between aromatic groups may be vital to energy storage under harsh conditions. For instance, we observe that ε_*r*_ and *E*_b_ in polyimide (PI) with no ether linkage remain nearly unchanged upon proton irradiation (Figures S10–S13, Supporting Information). By contrast, poly(ether ether ketone) (PEEK) with a single ether bond enables simultaneously improved ε_*r*_ and *E*_b_ (Figures S14–S17, Supporting Information). As PEEK shows only one ether bond and a higher modulus (Figure S18, Supporting Information), the number of polar states might be smaller than PEI, and the presence of high modulus makes large angular distortion in PEEK energetically more difficult. As a result, the energy storage properties of irradiated PEEK at 150 °C are considerably lower than PEI (Figure S17, Supporting Information). For instance, ε_*r*_ of PEEK is ≈4.1 irradiated at *x* = 20 and 40 Mrad compared to that of 3.2 at *x* = 0 Mrad for pristine PEEK. Meanwhile, *U*_d_ at *η* > 95% is 3.6 J cm^−3^ at 20 Mrad which is about four times as large as that (0.9 J cm^−3^) of pristine PEEK measured at 150 °C (Figure S17, Supporting Information). These results suggest that the design strategy developed in this work is generally applicable to aromatic polymers with ether bonds. In addition, we find that proton irradiation plays a negligible role in tuning the glass transition temperature (Figures S19 and S20, Supporting Information) and the decomposition temperature (Figures S21–S23, Supporting Information) based on differential scanning calorimetry (DSC) and thermogravimetric analysis (TGA) measurements, suggesting the absence of the severe destructions of polymer chains and complex chemical reactions introduced by proton irradiation.

The inversely correlated ε_*r*_ and *E*_b_ has been frequently overcome by various strategies.^[^ ^5^, ^37^, ^38^ ^]^ For instance, in dielectric ceramics, the design of the local diverse polarization^[^ ^37^ ^]^ and high‐entropy strategy^[^ ^38^ ^]^ have been used to address the trade‐off between ε_*r*_ (or polarization) and *E*_b_. A recent irradiation study in semicrystalline relaxor ferroelectric polymers shows that proton irradiation enables synergistic improvement in dielectric energy storage.^[^ ^39^, ^40^ ^]^ In particular, the formation of irradiation‐induced reaction brings a previously unidentified high‐entropy superparaelectric phase with markedly enhanced ε_*r*_ and polarization.^[^ ^39^, ^40^ ^]^ While the polar states in ferroelectric polymers can be described using various techniques,^[^ ^32^, ^33^, ^41^ ^]^ the counterparts in aromatic polymers remain at a very early stage, mainly owing to the challenge in depicting the amorphous structure with complex morphology and structural disorder.^[^ ^42^, ^43^, ^44^, ^45^, ^46^, ^47^ ^]^

Our results are also in line with a recent blend approach, which shows the presence of dense twisted polymer morphology enabling both enhanced dielectric constant and breakdown field, and thus resulting in markedly enhanced energy storage properties at high temperatures.^[^ ^48^ ^]^ Both the previous study and current work address the essential role of polymer morphology in tuning of dielectric energy storage properties. The reduced interchain distance caused by irradiation may not necessarily indicate an increase in the intramolecular charge transport owing to the presence of angular distortions instead of planar architectures. The dense chain packing is crucial to retain the large breakdown field by preventing the electromechanical failure, which typically occurs during the breakdown process. In our case, although the formation of twisted chain morphology needs to be verified by both theoretical and experimental efforts, this unique morphology is compatible with the main experimental findings, allowing both reduced interchain distance and stronger local heterogeneity, which may lead to the concurrent generation of a large dielectric constant and breakdown field. Further studies on the detailed structure of distorted chain morphology are highly desirable to reveal the correlation between local chain structure and energy storage properties. Given that a greatly enhanced dielectric constant has been extensively achieved in various dilute nanocomposites, whereas the local polymer chain nanostructures are of importance,^[^ ^29^ ^]^ our results widen the spectrum of high‐dielectric‐high‐temperature polymer‐based dielectric materials through proton irradiation, inspiring future development in this field from both scientific understanding and practical application.

## 3 Conclusion

In this work, we have demonstrated that proton irradiation can be used to drive the high‐dielectric and high‐insulating state with dense chain packing and enhanced polarizability in aromatic polymers, which provides a solution to address the long‐standing challenge in the field of high‐temperature dielectric energy storage. We show that the critical linkage bond‐capacitive property relationship may be generic to aromatic polymers with ether bonds, which enables superior energy storage at high temperatures. Differing from the conventional composition approach, irradiation modification offers postprocessing opportunities to optimize energy storage properties under harsh conditions.

## 4 Experimental Section

### Polymer Films

PEI and Matrimid 5218 PI were purchased from Polyk Technologies. 1‐Methyl‐2‐pyrrolidinone (NMP, anhydrous, 99.8%) was purchased from Sigma–Aldrich. All materials were used without further modifications. PEEK films with a thickness of 10 µm were purchased from Polyk Technologies.

PEI and PI films were fabricated by the solution casting method. 0.8 g of PEI or PI pellets were dissolved in 20 mL of NMP to obtain a concentration of 40 mg mL^−1^ solution, followed by stirring at 60 °C for 12 h. Then, the solution was cast on a pre‐cleaned glass plate and dried at 80 °C for 8 h. Afterward, the polymer films were annealed at 120 °C for 1 h followed by heating to 150 °C and annealing for 1 h, which was heated to 200 °C for 1 h. After that, the temperature decreased slowly to room temperature. Finally, the films were peeled off from the glass substrate and dried at 150 °C in a vacuum to remove residual solvent. The thickness of the films was ≈10 µm.

### Proton Irradiation

The high‐energy beam with a proton energy of 20 MeV was directed vertically to the surface of the polymer films (size: 1 × 1 cm^2^) with the proton flux and fluence of 2 × 10^8^ p cm^−2^ s^−1^ and 4×10^10^ p cm^−2^, respectively. Varied irradiation doses (*x* = 10, 20, and 40 Mrad) were used for the irradiation experiments.

### Characterization

FTIR was performed with a Thermo Scientific Nicolet iS50R spectrometer with a wavenumber range from 400 to 4000 cm^−1^. XRD patterns were obtained by an X'pert3 Powder (PANalytical B.V.). AFM‐IR results were obtained by a Bruker Anasys nanoIR3 system in a tapping mode. The chemical mapping of the polymer composition was performed with a laser at a specific wavenumber (i.e., 1073 cm^−1^ for symmetric stretching of ether bonds). The local spectra were collected with a resolution of 2 cm^−1^. UV–vis absorption spectra were measured by a 46 SolidSpec‐3700 spectrophotometer, ranging from 200 to 800 nm.

### Thermal and Mechanical Analysis

TGA measurement was carried out by a Pyris1 TGA meter (PerkinElmer Instruments), ranging from 30 to 800 °C at a heating rate of 10 °C min^−1^ under a nitrogen atmosphere. DSC measurement was carried out by a Netzsch STA449F3 analysis meter (PerkinElmer Instruments) at a heating rate of 10 °C min^−1^ in a nitrogen atmosphere. The Young's modulus was derived from the stress–strain curve measured by a Discovery DMA850 (Waters).

### Electrical Measurement

Before measuring the electrical and dielectric properties, gold electrodes were sputtered (Quorum, Q150RS) on both sides of films with a typical thickness of 50 nm. Dielectric constant and loss versus frequency from 100 Hz to 100 MHz were tested by an E4980A precision LCR meter (Keysight Technologies). Temperature‐dependent dielectric properties were also measured by an E4980A precision LCR meter with a heating rate of 1 °C min^−1^ in an oven (Sun Electronics). Leakage currents and TSDC currents were measured by a Keithley 6517B amperemeter, and the voltage and temperature were controlled by a Trek DCQ‐201B and an oven (Sun Electronics), respectively. The leakage current was fitted by the relationship $J=2q\lambda nv\;\;\exp\left[\frac{q\lambda E}{2k_{B}T}-\frac{E_{a}}{k_{B}T}\right]$, where *J* is the current density, *q* is the electronic charge, *n* is the electron concentration in the conduction band of the dielectric, *ν* is the frequency of thermal vibration of electrons at trap sites, *T* is the temperature, and *E*_a_ is the activation energy. All samples for TSDC measurements were polarized under a DC electric field of 50 MV m^−1^ for 30 min at 200 °C, and then quickly cooled to 50 °C with the electric field of 50 MV m^−1^ applied. After removal of the electric field, the samples were immediately short‐circuited and heated to 250 °C at a heating rate of 3 °C min^−1^. During the heating process, the current was recorded. The electrode diameter for dielectric measurement, leakage current, and TSDC current tests was 4 mm. *P*‐*E* loops were measured by a modified Sawyer–Tower circuit. The samples were placed in temperature‐resistant silicone oil in a glass container, which was placed on the top of a hot plate. The temperature was tuned by the underlying hot plate. Breakdown strength was measured by a dielectric breakdown system (PolyK) with a TREK 610B instrument at a voltage rising rate of 600 V s^−1^, and 15 different data were recorded and analyzed by the two‐parameter Weibull statistic *p*(*E*) = 1‐exp(‐(*E*/*E*_b_)*^β^*), where *p*(*E*) depicts the cumulative probability of dielectric failure, *E*_b_ refers to the Weibull breakdown strength corresponding to 63.2% probability of breakdown, and *β* describes the scattering of the experimental data.

### DFT Calculations

The calculations on the structure of PEI were performed by DFT calculations using the GAUSSIAN program package^[^ ^49^ ^]^ with B3LYP/6‐311+G(d,p) level of theory.^[^ ^50^ ^]^ The DFT‐D3 method with Becke–Johnson damping function was adopted for the dispersion energy correction.^[^ ^51^, ^52^ ^]^ After the geometry optimization, the molecule was presented in Figure 1a. In Figure 1a, dihedral angles and planes were indicated, i.e., the dihedral angles *θ*_1_ to *θ*_6_, with *θ*_1_ relating to benzene plane 1 (P_1_) and C‐O‐C plane 1 (P_COC1_), *θ*_2_ referring to benzene plane 2 (P_2_) and C‐O‐C plane 1 (P_COC1_), etc. Furthermore, rigid potential energy surface (PES) scans, indicating the dihedral angle rotation to enable energy gain, were performed, which consisted of single‐point energy evaluations over a rectangular grid (37×37). The optimized dihedral angles from *θ*_1_ to *θ*_6_ were 12.6°, 70.4°, 130.1°, 129.9°, 116.5°, and −15.1°, respectively. During PES scans, the dihedral angle *θ*_1_ varied from *θ*_1_‐90° to *θ*_1_+90°, with a step of 5° (i.e., 37 values for each dihedral angle), which was repeated for other dihedral angles (*θ*_2_ to *θ*_6_).

The electric dipole moments (*µ*_tot_ = *µ*_elec_ + *µ*_nuc_) of different molecular geometries, consisting of the electronic dipole moment (*µ*_elec_), derived from the integration of electron density over the entire spatial domain, and the nuclear dipole moment (*µ*_nuc_), determined by the positions and charges of atomic nuclei, could be calculated by the GAUSSIAN package. In particular, the angles *θ*_1_, *θ*_3_, and *θ*_5_ were fixed at 0°, −5°, 10° and −15° whereas *θ*_2_, *θ*_4_, *θ*_6_ were increased from 0° to 90° at a step of 5°.

## Conflict of Interest

The authors declare no conflict of interest.

## Author Contributions

C.Y.L., H.X.G., and Y.T.G. contributed equally to this work. L.C., Yang Li, Yang Liu, and H.M.Z. conceived the idea and designed the research. C.Y.L. prepared polymer films. C.Y.L., H.X.G., and Y.T.G. collected XRD, FTIR, UV–vis absorption spectra, DSC, DMA, and TGA data. C.Y.L., Y.Q.L., Y.Q.W., L.C., and H.Y.W. performed electrical measurements. C.Y.L. and Y.‐T.L. performed AFM‐IR measurements. C.Y.L. performed DFT calculations. L.C., Yang Li, Yang Liu, and H.M.Z. wrote the manuscript with feedback from all authors.

## Supporting information



Supporting Information

## Data Availability

The data that support the findings of this study are available from the corresponding author upon reasonable request.
