# Supplementary material for: Ultrahigh High‐temperature Capacitive Energy Storage Via Proton Irradiation
Source: Adv Sci (Weinh). 2025 Nov 30;13(9):e22035. doi: 10.1002/advs.202522035 (PMC12904005; doi:10.1002/advs.202522035)
Supplement: Supplementary file 1 — Supporting Information [file ADVS-13-e22035-s001.docx]

Supporting Information

**Ultrahigh high-temperature capacitive energy storage via proton irradiation**

*Chenyi Li^#^, Hanxiao Gao^#^, Yutie Gong**^#^, Yen-Ting Lin,* *Yuquan Liu, Yuanqi Wang, Lan Chen, Hangyao Wu, Ling Cheng^*^, Yang Li^*^**, Yang Liu**^*^, Huamin Zhou^*^*

Email: lawliet_cheng@163.com; liyang@hust.edu.cn; yliu1319@hust.edu.cn; hmzhou@hust.edu.cn

**Table of Contents**

**Figs. S1-S23 2-24**

**Tables S1-S2 25-26**

**References S1-S15 27-28**

**Fig. S1.** **Sketch of *P*-*E* loops used to evaluate *U*_d_ and *η*.**

**Fig. S2. Sketch of chemical structures of PEI (a), PI (b) and PEEK (c).**

**Fig. S3. Dipole moment under varied *θ*_2_, *θ*_4_, and *θ*_6_ (*θ*_1_****=*θ*_3_=*θ*_5_=-15^o^).**

**Fig. S4. FTIR spectra in pristine and irradiated PEI.**

**Fig. S5. TSDC results in pristine and irradiated PEI.**

**Fig. S6. The remanent polarization *P*_r_ (a), the maximum polarization *P*_m_ (b) and the polarization change Δ*P* (c) in pristine and irradiated PEI.**

**Fig. S7. *P*-*E* loops in irradiated PEI at 150 °C. a**, *x*=10 Mrad. **b**, *x*=40 Mrad.

**Fig. S8. *P*-*E* loops in irradiated PEI at 150 °C.** **a**, *x*=0 Mrad. **b**, *x*=20 Mrad.

**Fig. S9.** ***U*_d_ and** ***η* in irradiated PEI at 150 °C. a**, *x*=10 Mrad. **b**, *x*=40 Mrad.

**Fig. S10.** **Dielectric constant (a) and loss (b) in irradiated PI.**

**Fig. S11.** **Temperature dependence of dielectric constant (a) and loss (b) in irradiate PI.**

**Fig. S12. *P*-*E* loops in irradiated PI at 150 °C. a**, *x*=0 Mrad. **b**, *x*=10 Mrad. **c**, *x*=20 Mrad. **d**, *x*=40 Mrad.

**Fig. S13. *U*_d_ (a) and *η* (b) in irradiated PI at 150 °C.**

**Fig. S14. Dielectric constant (a) and loss (b) in irradiated PEEK.**

**Fig. S15. Summary on dielectric constant and loss as a function of irradiation dose in irradiated PEEK.**

**Fig. S16. *P*-*E* loops in irradiate PEEK at 150 °C. a**, *x*=0 Mrad. **b**, *x*=10 Mrad. **c**, *x*=20 Mrad. **d**, *x*=40 Mrad.

**Fig. S17. *η* (a) and *U*_d_ (b) in irradiated PEEK at 150 °C.**

**Fig. S18. Stress-strain curves in irradiated PEEK.**

**Fig. S19. DSC scans near the glass phase transition temperature in irradiated PEI.** **a**, *x*=0 Mrad. **b**, *x*=10 Mrad. **c**, *x*=20 Mrad. **d**, *x*=40 Mrad.

**Fig. S20. DSC scans in irradiated PEEK.** **a**, *x*=0 Mrad. **b**, *x*=10 Mrad. **c**, *x*=20 Mrad. **d**, *x*=40 Mrad.

**Fig. S21. TGA results in irradiated PEI.**

**Fig. S22. TGA results in irradiated PI.**

**Fig. S23. TGA results in irradiated PEEK.**

**Table S1. Comparison of maximum *U*_d_ at above 95% *η* between this work and the state-of-the-art results at 150 ^o^C**

| Dielectric Materials | Dielectric constant | *U*_d_ @*η*≥95%  (J cm^-3^) | Electric field  (MV m^-1^) | Frequency  (Hz) | Ref. |
| --- | --- | --- | --- | --- | --- |
| **Proton-irradiated PEI (20 Mrad)** | **6.0** | **6.9** | **510** | **100** | **This work** |
| **Synthesized Polymers** |  |  |  |  |  |
| PSBNP-co-PTNI_0.02_ | 4.3 | 3.9 | 450 | 10 | S1 |
| polysulfate P_3_ | 3.3 | 3.1 | 420 | 100 | S2 |
| o-POFNB | 2.9 | 2.7 | 430 | 100 | S3 |
| ht-PEKNA | 5.3 | 2.2 | 350 | 10 | S4 |
| NH_2_-POSS-PI | 3.0 | 3.2 | 450 | 100 | S5 |
| PEEK-CN | 4.4 | 1.2 | 250 | 200 | S6 |
| **All-organic polymers** |  |  |  |  |  |
| PEI/PBCM | 3.4 | 3.7 | 470 | 10 | S7 |
| PC/ITIC-Cl | 3.6 | 3.8 | 480 | 100 | S8 |
| F-PI/PBCM | 3.0 | 3.6 | 520 | 100 | S9 |
| **Other polymers** |  |  |  |  |  |
| PEI/PI blend | 3 | 0.5 | 180 | 10 | S10 |
| Cross-linked CS-ODA | 3.5 | 2.2 | 350 | 100 | S11 |
| **Polymer nanocomposites** |  |  |  |  |  |
| PEEU/Al_2_O_3_ | 7.4 | 2.5 | 250 | 10 | S12 |
| BCB/BNNS | 3.1 | 0.8 | 250 | 10 | S13 |
| PEI/SiO_2_ | 3.9 | 1.5 | 300 | 100 | S14 |
| **Multilayer structure** |  |  |  |  |  |
| Al_2_O_3_/P_3_/Al_2_O_3_ | 3.3 | 4.6 | 505 | 100 | S2 |
| Al_2_O_3_/PEI/Al_2_O_3_ | 3.2 | 2.6 | 430 | 10 | S15 |

**Table S2. Comparison of maximum *U*_d_ between this work and the state-of-the-art results at 150 ^o^C**

| Dielectric Materials | Maximum *U*_d_  (J cm^-3^) | Electric field  (MV m^-1^) | Frequency  (Hz) | Ref. |
| --- | --- | --- | --- | --- |
| **Proton-irradiated PEI (20 Mrad)** | **11.3** | **700** | **100** | **This work** |
| **Synthesized Polymers** |  |  |  |  |
| PSBNP-co-PTNI_0.02_ | 10.4 | 760 | 10 | S1 |
| polysulfate P_3_ | 6.0 | 600 | 100 | S2 |
| o-POFNB | 8.3 | 800 | 100 | S3 |
| ht-PEKNA | 3.1 | 400 | 10 | S4 |
| NH_2_-POSS-PI | 5.8 | 610 | 100 | S5 |
| PEEK-CN | 4.0 | 450 | 200 | S6 |
| **All-organic polymers** |  |  |  |  |
| PEI/PBCM | 4.8 | 550 | 10 | S7 |
| PC/ITIC-Cl | 8.8 | 650 | 100 | S8 |
| F-PI/PBCM | 6.4 | 700 | 100 | S9 |
| **Other polymers** |  |  |  |  |
| PEI/PI blend | 1.7 | 500 | 10 | S10 |
| Cross-linked CS-ODA | 7.0 | 650 | 100 | S11 |
| **Polymer nanocomposites** |  |  |  |  |
| PEEU/Al_2_O_3_ | 10.6 | 600 | 10 | S12 |
| BCB/BNNS | 2.4 | 420 | 10 | S13 |
| PEI/SiO_2_ | 6.3 | 620 | 100 | S14 |
| **Multilayer structure** |  |  |  |  |
| Al_2_O_3_/P_3_/Al_2_O_3_ | 8.6 | 750 | 100 | S2 |
| Al_2_O_3_/PEI/Al_2_O_3_ | 4.0 | 550 | 10 | S15 |

**References**

[S1] J. Chen, Y. Zhou, X. Huang, C. Yu, D. Han, A. Wang, Y. Zhu, K. Shi, Q. Kang, P. Li, P. Jiang, X. Qian, H. Bao, S. Li, G. Wu, X. Zhu, Q. Wang, Ladderphane Copolymers for High-Temperature Capacitive Energy Storage. *Nature* **2023**, *615*, 62.

[S2] H. Li, B. S. Chang, H. Kim, Z. Xie, A. Laine, L. Ma, T. Xu, C. Yang, J. Kwon, S. W. Shelton, L. M. Klivansky, V. Altoe, B. Gao, A. M. Schwartzberg, Z. Peng, R. O. Ritchie, T. Xu, M. Salmeron, R. Ruiz, K. B. Sharpless, P. Wu, Y. Liu, High-Performing Polysulfate Dielectrics for Electrostatic Energy Storage under Harsh Conditions. *Joule* **2023**, *7*, 95.

[S3] A. A. Deshmukh, C. Wu, O. Yassin, A. Mishra, L. Chen, A. Alamri, Z. Li, J. Zhou, Z. Mutlu, M. Sotzing, P. Rajak, S. Shukla, J. Vellek, M. A. Baferani, M. Cakmak, P. Vashishta, R. Ramprasad, Y. Cao, G. Sotzing, Flexible Polyolefin Dielectric by Strategic Design of Organic Modules for Harsh Condition Electrification. *Energy Environ. Sci.* **2025**, *15*, 1307.

[S4] D. Xu, W. Xu, T. Seery, H. Zhang, C. Zhou, J. Pang, Y. Zhang, Z. Jiang, Rational Design of Soluble Polyaramid for High-Efficiency Energy Storage Dielectric Materials at Elevated Temperatures. *Macromol. Mater. Eng.* **2020**, 305, 1900820.

[S5] J. Dong, L. Li, P. Qiu, Y. Pan, Y. Niu, L. Sun, Z. Pan, Y. Liu, L. Tan, X. Xu, C. Xu, G. Luo, Q. Wang, H. Wang, Scalable Polyimide-Organosilicate Hybrid Films for High-Temperature Capacitive Energy Storage. *Adv. Mater.* **2023**, *35*, 2211487.

[S6] W. Huang, T. Ju, R. Li, Y. Duan, Y. Duan, J. Wei, L. Zhu, High-κ and High-Temperature Dipolar Glass Polymers Based on Sulfonylated and Cyanolated Poly(Arylene Ether)s for Capacitive Energy Storage. *Adv. Electron. Mater.* **2023**, *9*, 2200414.

[S7] C. Yuan, Y. Zhou, Y. Zhu, J. Liang, S. Wang, S. Peng, Y. Li, S. Cheng, M. Yang, J. Hu, B. Zhang, R. Zeng, J. He, Q. Li, Polymer/Molecular Semiconductor All-Organic Composites for High-Temperature Dielectric Energy Storage. *Nat. Commun.* **2020**, *11*, 3919.

[S8] Y. Zhou, Y. Zhu, W.Xu, Q. Wang, Molecular Trap Engineering Enables Superior High-Temperature Capacitive Energy Storage Performance iin All‐Organic Composite at 200 °C. *Adv. Energy Mater.* **2023**, *13*, 2203961.

[S9] W. Ren, M. Yang, L. Zhou, Y. Fan, S. He, J. Pan, T. Tang, Y. Xiao, C. W. Nan, Y. Shen, Scalable Ultrathin All-Organic Polymer Dielectric Films for High-Temperature Capacitive Energy Storage. *Adv. Mater.* **2020**, *34*, 2207421.

[S10] Q. Zhang, X. Chen, B. Zhang, T. Zhang, W. Lu, Z. Chen, Z. Liu, S. H. Kim, B. Donovan, R. J. Warzoha, E. D. Gomez, J. Bernholc, Q. M. Zhang, High-Temperature Polymers wwith Record-High Breakdown Strength Enabled by Rationally Designed Chain-Packing Behavior in Blends. *Matter* **2021**, *4*, 2448.

[S11] Z. Pan, L. Li, L. Wang, G. Luo, X. Xu, F. Jin, J. Dong, Y. Niu, L. Sun, C. Guo, W. Zhang, Q. Wang, H. Wang, Tailoring Poly(Styrene-*co*-maleic anhydride) Networks for All-Polymer Dielectrics Exhibiting Ultrahigh Energy Density and Charge-Discharge Efficiency at Elevated Temperatures. *Adv. Mater.* **2023**, *35*, 2207580.

[S12] T. Zhang, X. Chen, Y. Thakur, B. Lu, Q. Y. Zhang, J. Runt, Q. M. Zhang, A Highly Scalable Dielectric Metamaterial with Superior Capacitor Performance over a Broad Temperature. *Sci. Adv.* **2020**, *6*, eaax6622.

[S13] Q. Li, L. Chen, M. R. Gadinski,S. Zhang,G. Zhang,U. Li, E. Iagodkine, A. Haque, L.-Q. Chen, N. Jackson, Q. Wang, Flexible High-Temperature Dielectric Materials from Polymer Nanocomposites. *Nature* **2015**, *523*, 576.

[S14] Y. Zhou, Q. Li, B. Dang, Y. Yang, T. Shao, H. Li, J. Hu, R. Zeng, J. He, Q. Wang, A Scalable, High-Throughput, and Environmentally Benign Approach to Polymer Dielectrics Exhibiting Significantly Improved Capacitive Performance at High Temperatures. *Adv. Mater.* **2018**, *30*, 1805672.

[S15] S. Cheng, Y. Zhou, Y. Li, C. Yuan, M. Yang, J. Fu, J. Hu, J. He, Q. Li, Polymer Dielectrics Sandwiched by Medium-Dielectric-Constant Nanoscale Deposition Layers for High-Temperature Capacitive Energy Storage. *Energy Stor. Mater.* **2021**, 42, 445.
